# Contamination within trials of community-based public health interventions: lessons from the HENRY feasibility study

**DOI:** 10.1186/s40814-021-00805-3

**Published:** 2021-03-26

**Authors:** Elizabeth Stamp, Holly Schofield, Victoria Laurina Roberts, Wendy Burton, Michelle Collinson, June Stevens, Amanda Farrin, Harry Rutter, Maria Bryant

**Affiliations:** 1 School of Sport, Exercise and Health Sciences, Loughborough University, Loughborough, LE11 3TU UK; 2 Clinical Trials Research Unit, Leeds Institute of Clinical Trials Research, University of Leeds, Leeds, LS2 9JT UK; 3 School of Medicine, University of Leeds, Leeds, LS2 9JT UK; 4 Department of Health Sciences, University of York, York, YO10 5DD UK; 5 Department of Nutrition, Gillings School of Public Health, University of North Carolina, Chapel Hill, 27599 USA; 6 Department of Social & Policy Sciences, University of Bath, Bath, BA2 7AY Somerset UK; 7 Hull York Medical School, University of York, York, YO10 5DD UK

**Keywords:** Contamination, Public health, Childhood, Community, Obesity, Randomised control trial

## Abstract

**Introduction:**

Contamination occurs when participants allocated to trial control arms receive elements of the active intervention. Randomisation at cluster level, rather than individual level, may reduce or eliminate contamination, avoiding the dilution of intervention effectiveness that it may cause. However, cluster randomisation can result in selection bias and may not be feasible to deliver. We explored the extent of contamination in a qualitative study nested within a feasibility study of HENRY (Health, Exercise and Nutrition for the Really Young); a UK community-based child obesity prevention programme. We aimed to determine the nature and impact of contamination to inform a larger planned trial and other trials in community based public health settings.

**Method:**

We invited participants to take part in the nested qualitative study who were already involved in the HENRY feasibility study. Semi-structured interviews/focus groups were conducted with children’s centre managers (*n*=7), children’s centre staff (*n*=15), and parents (*n*=29). Data were transcribed and analysed using an integrative approach. First, deductively organised using a framework guided by the topic guide and then organised using inductive thematic analysis.

**Results:**

Potential for contamination between treatment arms was recognised by all stakeholder groups. Staff within the intervention centres presented the greatest risk of contamination, predominantly because they were often asked to work in other children centre’s (including control group centres). ‘Sharing of best practice’ by staff was reported to be a common and desirable phenomenon within community based settings. Parental sharing of HENRY messages was reported inconsistently; though some parents indicated a high degree of knowledge transfer within their immediate circles.

**Conclusions:**

The extent of contamination identified has influenced the design of a future effectiveness trial of HENRY which will be clustered at the centre level (with geographically distinct clusters). The common practice of knowledge sharing amongst community teams means that this clustering approach is also likely to be most suitable for other trials based within these settings. We provide recommendations (e.g. cluster randomisation, training intervention facilitators on implications of contamination) to help reduce the impact of contamination in public health intervention trials with or without clustering, whilst enabling transfer of knowledge where appropriate.

**Trial registration:**

ClinicalTrials.gov Identifier NCT03333733 registered 6th November 2017

## Key messages

- **What uncertainties existed regarding the feasibility?**

Trials to test the effectiveness of community-based childhood obesity prevention interventions are needed considering the high obesity rates at school entry. However, it is uncertain whether the information provided as part of community-based interventions is shared with the control group in RCTs. We wanted to explore the extent and implications of contamination in the HENRY feasibility study and identify suitable strategies to reduce contamination.

- **What are the key feasibility findings?**

This research identified that there was contamination between treatment arms, as recognised by all stakeholder groups that were interviewed. Staff appears likely to pose the greatest risk of contamination within community-based settings. Following discussions within the research team and the trial steering committee, we have identified strategies to reduce the extent of contamination in the definitive trial, as well as other RCT’s in community-based settings.

- **What are the implications of the feasibility findings for the design of the main study?**

This research has been important during the development of the main trial. A key finding for our future trial was that staff posed the greatest risk of contamination. Therefore, we will ensure that centre clusters are geographically distinct, and we will train all staff about the need to withhold knowledge from control centres.

## Background

Childhood obesity is a major and growing public health problem, even in the early years, with almost 10% of children starting school with obesity in the UK [1]. Children with obesity can experience physiological and psychological health implications, which can continue into adulthood [2, 3]. Obesity also presents financial implications, with an estimated £6 billion attributed to obesity and overweight related ill-health annually in England alone [4]. In the UK, local governments commonly commission community-based prevention programmes as one strategy to meet the national target of reducing childhood obesity prevalence by 50% before 2030 [5]. Programme effectiveness is an important factor when deciding which programmes to commission and implement [6]; however, evidence is often lacking to support this [7].

Trials of complex interventions such as those delivered to groups within community settings carry a risk of contamination, where participants in the control arm passively or actively receive some of the intervention [8]. In particular, educational interventions for behaviour change are susceptible to contamination as it is challenging to confine information solely to the intervention group [8, 9]. Contamination can occur through different routes, including intervention facilitators (e.g. staff moving between sites), participants (e.g. exchanging information between control and intervention arms), or dissemination of the programme (e.g. when participants randomised into the control arm obtain further information about the trial) [10, 11]. While wider reach for an intervention may be helpful in terms of dissemination, contamination presents a challenge to researchers attempting to assess intervention effectiveness [9]. Contamination may lead to reductions in observed effect sizes, potentially resulting in rejection of an effective intervention [8, 12]. Randomisation at the cluster level can reduce the impact of contamination [9, 13], but risks introducing selection bias if participant recruitment occurs post-randomisation [14]. In addition, clustering has an impact on trial feasibility, often requiring larger sample sizes and a greater number of sites. It has been argued that these factors may outweigh the benefits of using cluster randomisation to reduce contamination [9]. Other methodologies may also be applied to reduce contamination in trials, including recruitment of participants prior to the randomisation of clusters [9, 15].

The extent and implications of contamination in trials assessing the effectiveness of community-based public health interventions are not fully understood [16], particularly for parent and child interventions [10]. We therefore aimed to investigate contamination within a randomised feasibility study of HENRY; a UK community-based childhood obesity prevention programme [17] [Bryant et al., submitted at same time to Pilot and Feasibility Studies].

In order to support the design of a future effectiveness trial and provide wider evidence to support the design of similar trials in this setting, this study looked to identify sources of contamination in the HENRY study, the extent to which contamination occurred, factors that increased the risk of contamination, and the implications of contamination.

## Methods

### Design

We used qualitative methods (interviews and focus groups) to explore contamination within the HENRY feasibility study [17] [Bryant et al., submitted at same time to Pilot and Feasibility Studies]. The feasibility study was an NIHR funded, multicentre, two-arm, cluster RCT. The protocol is reported elsewhere [17]. In brief, the study aimed to recruit 120 parents across 12 children’s centres in two local authorities (governments). Primary objectives were to assess the feasibility of recruiting local authorities, centres and parents; to test processes and time required to train and certify intervention staff; explore HENRY commissioning processes; and determine the feasibility of trial procedures. It also aimed to identify potential sources and the associated impact of contamination. This final objective to explore contamination was delivered within a nested qualitative study and is reported here. Our proposed qualitative methods were initially piloted within a local authority that was not part of the feasibility study. As there were few changes to the protocol as a result of the pilot (and assumption that being part of the feasibility study would not influence findings), data from the pilot stage are included here. Research was approved by the University of Leeds School of Medicine Research Ethics Committee (MREC: 16-107). The HENRY feasibility study (incorporating this qualitative research) was registered on clinicaltrials.gov (#NCT03333733).

#### Description of the HENRY intervention

HENRY is an 8-week parenting programme delivered across approximately 40 local authorities in children centres across the UK. Details of the programme are provided elsewhere [17–19]. In brief, groups of approximately eight to ten parents of pre-school children attend weekly sessions within community settings. The programme aims to provide parents with the skills and knowledge that are required to encourage healthy lifestyles in preschool children and their families. Topics covered in the programme include eating habits, balancing healthy meals and snacks, child appropriate portion sizes, emotional wellbeing, parenting skills and activity [20]. Further information on the HENRY programme can be found here: https://henry.org.uk/.

### Recruitment

Pilot work in a local authority that did not participate in the feasibility study recruited parents and staff from a children’s centre that delivered the HENRY programme through invitations/promotions within the centres. For the main nested study, feasibility study participants were recruited during the follow-up stage of data collection. We sought to recruit key stakeholders who were involved in the HENRY feasibility study [17] including (1) children’s centre managers, (2) children’s centre staff who recruited parents to the feasibility study, (3) children’s centre staff who delivered the HENRY intervention, and (4) parent participants recruited to the feasibility study. A sampling framework was used to ensure representation from both local authorities and each arm of the study.

#### Recruitment of children’s centre staff and managers

All children centre managers and staff who had been involved in the HENRY feasibility study (e.g. parent recruitment, HENRY intervention delivery) were invited to take part in interviews via email by the research team in March 2019. Three e-mail reminders were sent to those who did not reply at two week intervals.

#### Recruitment of parents

Following the pilot phase, parents were initially invited to take part in focus groups via email in September 2018. However, there was low attendance at the first two focus groups (two participants at each focus group out of 36 invited parents), and the protocol was therefore amended so that the remaining parents were recruited to take part in telephone interviews instead. Potential participants who did not respond were contacted again via email and then via telephone up to three times. Parents received a £10 incentive for their participation.

### Data collection

After the pilot phase, researchers (HS, WB) conducted one focus group in each local authority at a children’s centre. Thereon, telephone interviews were conducted by two researchers (HS, ES). Written informed consent was obtained prior to commencing the pilot interviews and focus groups, and telephone consent was obtained prior to commencing the telephone interviews. Focus groups and interviews were led by topic guides which focused on sharing of healthy messages between networks (e.g. families, friends and children’s centre staff), both in general and specific to the HENRY programme, and the ways in which the messages were shared. Topic guides were developed to gain an understanding of the lived experiences of participants [21] and to provide a rich and detailed account of their experiences sharing healthy messages and HENRY programme content more specifically [22]. Data collection continued until the interviewer deemed data saturation had been reached [23].

### Data analysis

Data management and coding was conducted in NVivo [24]. An integrative approach was used [25]; initially, data were deductively organised using a framework guided by the topic guide. Data were then thematically analysed inductively to enable the emergence of new themes within the categories in the framework [25, 26]. To establish trustworthiness of the data [26], transcripts were transcribed to gain a feeling of the participant accounts, and two researchers discussed and agreed on their perceptions of the participant experience. In addition, 10% of the transcripts were independently coded, and discussions were used to resolve any inconsistencies. This process was done iteratively during data collection so that emerging findings could be discussed and expanded if necessary in subsequent interviews. Discussions between the research team enabled the development of contamination risk factor categories. The level of risk was based on the likelihood (frequency that the behaviour was reported) and impact (the implications of the behaviour on contamination). Behaviours were then allocated to one of four risk factor categories: high risk (high impact, high likelihood), medium-high risk (high impact, low likelihood), medium-low risk (low impact, high likelihood) and low risk (low impact, low likelihood). Strategies that could reduce risk factors for contamination were developed through discussions within the research team and parent advisory group meetings.

## Results

In total, 51 participants took part in the nested study from three local authorities, including nine parents across two focus groups in the pilot work and four parents from two focus groups within the feasibility study (Table 1).

**Table 1:** Summary of participants

Data collection	***n***	Description (including recruitment source)	
		Pilot phase recruitment	Feasibility study recruitment
**Focus groups**			
Parents	13	4 x attended HENRY; 5 x did not attend HENRY	3 x control; 1 x intervention
**Interviews**			
Parents	16	-	7 x control; 9 x intervention
Staff	15	2 x HENRY centres	4 x control; 4 x intervention 5 x control and intervention
Managers	7	1 x HENRY centre	2 x control; 1 x intervention 3 x control and intervention

There was a representation from both treatment arms (HENRY/non-HENRY in pilot work) for staff (control: 26.7%, intervention: 40%), managers (control: 25%, intervention: 37.5%) and parents (control: 52%, intervention 48%). A number of the staff (33.3%) and managers (37.5%) were found to work in both control and intervention centres. Focus groups lasted on average 45 min, and interviews lasted on average 22 min.

### Staff and manager perspectives

Staff appeared to pose the greatest risk of contamination within the feasibility study, mainly through face-to-face encounters, and less commonly through promotion of HENRY (social media and posters in centres). Staff working across multiple children centres, including both control and intervention centres, appeared to be key contributors to contamination within the feasibility study. This situation was a commonly reported and considered to be a positive way of sharing staff and knowledge. While one manager reported that staff at their centre made a conscious effort not to share the information learnt at their HENRY feasibility study training:

“They have been very mindful not to share anything of HENRY when they are at the other site. So they have made a conscious effort not to do that” (Manger, control & intervention centre), it was acknowledged that it was difficult not to incorporate the HENRY messages into everyday practice once learnt: “its hard because you can’t lose the learning that you have got can you?” (Manger, control & intervention centre).

Further, some staff also discussed how they would purposely incorporate HENRY messages into other programmes that they delivered as they felt that it would benefit parents, for example HENRY messages about portion sizes: “…the portion sizes, that’s obviously shared with the other centres as well, cos [because] it is really important cos the portion size of children was quite large. So we have embedded those kind of…in the other programmes as well.” (Staff, control & intervention centre).

Staff meetings at the district level presented further potential for contamination, as we learnt that staff from a number of centres met to discuss best practice and share ideas: *“*Although we work in different centres we all meet up every month for a full team meeting, we talk about what's going on in centres and it [HENRY] could be mentioned there.” (Staff, intervention centre).

Some children centre staff reported sharing HENRY messages at team meetings as they perceived it as helping others: “Yes obviously we have our health forums, our start well forums and things like that. Some of them haven’t even started HENRY. So giving that information out that is really useful for them.” (Staff, control and intervention centre).

Staff in the centres delivering HENRY also reported personally implementing behaviour change based on what they had learnt from attending HENRY training*:* “And like I say we practice what we preach and try and be a role model and we have healthy snacks, healthy things in our fridge and it makes us think more about what we are eating and the benefits to us of healthy eating and giving you more energy during the day because when you are doing owt [anything] you need more energy that you think. I think on the whole it’s had an impact on everybody. Everybody has opened their eye to like adapting things from HENRY into our daily lifestyle.” (Staff, intervention centre).

These behaviours may have been observed by parents attending centres, but the degree to which this could influence parental behaviours is uncertain.

Contamination occasionally occurred through the promotion of the HENRY programme to parents and via sharing of healthy messages that were learnt through attending HENRY training. This was variable. For example, a manager in one of the local authorities reported that centres did not use social media to share healthy advice, whereas the another manager from the other local authority admitted to using it for this purpose: “We have Facebook, so we use Facebook and our worker who puts on Facebook, she will put out a message once a month or something.” (Manager, control & intervention centre).

Most intervention centres reported that they advertised the HENRY programme using posters and display boards. Whilst the feasibility study attempted to limit this to intervention centres only, displays could be observed by any parents visiting from other centres:

“We have things like the HENRY display out. We have all the books… we have displays about portions and things like that. So really it’s all over the place. Sometimes they don’t even known [laughter] you’re telling them. It’s stuff they pick up” (Staff, Intervention centre).

### Parent perspectives

Parents appeared to present a smaller risk of contamination compared to children’s centre staff and managers. Control parents who were interviewed had limited knowledge about the HENRY programme prior to being recruited into the feasibility study: “Just that it was to do with healthy eating for the child and also the mother that was the brief I got from it.” (Parent, control centre).

Parents did not report investigating the HENRY programme or finding out more information; however, a small number did report that they changed their behaviour due to being recruited into the study and knowing that they would be weighed at follow up:

“Yeah I am now much more stricter on my diet than I was before. I try to because everybody wants to be happy with their weight.” (Parent, control centre)

Parents suggested that their choice of which children’s centre to attend was based predominantly on the distance that they had to travel. The majority said that they usually attended just one centre (thus reducing the potential of knowledge transfer between centres); however, a small number of parents also visited other centres or knew of others who attended multiple centres:

“…….. if you aren’t close it makes it very hard for parents to be able to attend. I think having knowledge of different children's centres that are also running the HENRY programme it might make it easier to schedule and be aware of how to get to things like that if it isn’t the centre you already go to. I think everyone that had come to our HENRY programme lived in a relatively close proximity to where we were attending” (Parent, intervention centre).

In terms of sharing of messages, parents said that, though they often discussed topics with other parents (or within other programmes provided by centres), these discussions were rarely reported to be specific to HENRY. Nevertheless, some reported conversations topics that were similar to those delivered in the HENRY programme (for example, portion size, healthy diet and physical activity):

“My friends, we have got quite a few in my friendship group that have got kids the same age. So we all kinda like share tips. Erm also, so at my daughter’s school like we’ve got friends, I’ve got mums that we all sort of just chat. So there is one of the mums who has recently just had a child as well, and so I have, so we sort of like you know you share tips, you talk about your experiences, what works, what doesn’t work.” (Parent, intervention centre).

We learnt that it was rare that parents would share advice without being prompted to do so. Instead, parents were most likely to discuss healthy behaviours when others asked others for advice: “With a parent if they’re struggling and they come to me for help and I've learnt it then I'll pass it on but I wouldn’t just go out and give it out in the street.” (Parent, intervention centre).

A small number of parents reported that they freely shared information if they thought it would provide benefit, as opposed to waiting to be asked for the advice: “I would share it definitely. I am somebody who would definitely share it. Especially with someone who I feel like would benefit from it. Or I could help their child or something I would definitely share it with them.” (Parent, intervention centre). This participant went on to specifically discuss the intervention and expressed that they would only share programme content with others who were attending the same centre: “Because you know, I am going to be honest with you, with the HENRY the only person I really discussed with HENRY was the other parents at the you know, the stay and play that didn’t go to it.” (Parent, intervention centre). Thus, the likelihood of parents sharing information outside of the centre was deemed to be low.

Some parents reported engaging with social media, predominantly to share existing posts. A small number of parents said they provided advice on social media or online forums*: “*I share on social media I'm part of a parenting support group on Facebook. So quite often we share little bits on there. If someone’s in some trouble we stick it up and there we all offer our advice.” (Parent, control centre).

### Impact of contamination on behaviour change

There appeared to be key factors that influenced the likelihood that hearing healthy messages led to parents changing their behaviour. The frequency of hearing advice about a specific topic was a key factor, with both parents and staff suggesting that parents usually need to hear health advice repeatedly before they changed their behaviours: “You get these parents that come and it doesn’t sink in first time. A lot of these parents they are vulnerable…. I think it’s good that they repeat” (Staff, intervention centre).

A further factor that influenced the likelihood of the information changing behaviours was the source of the information. For example, parents appeared to talk highly of, and trust, the advice of professionals (e.g., children centre staff and health visitors):

“Yes some of the health visitors again, our health visitors were really good. They would, you know, give you advice on what we were feeding our children. They would see things that you would give to them and say either oh that really good that you have given them that, or you should maybe wean them in this way. So yes it was definitely useful.” (Parent, control centre).

### Strategies to mitigate risk of contamination in public health trials

**Fig. 1:**
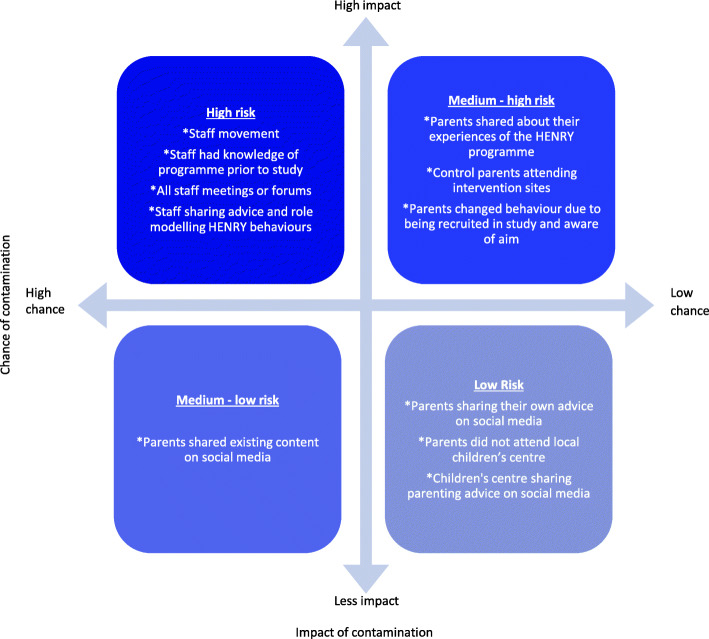
An overview of the behaviours that can lead to contamination and the associated risk

Data from this nested qualitative study led to the production of a contamination risk framework (Fig. 1). This was produced through discussions with the research team, the steering committee and our parent advisory group, leading to the development of categories indicating the groups’ judgement of likelihood of contamination, and its potential impact on behaviour changes (related to trial outcomes). For example, staff working at multiple centres was reported frequently and appeared to have a large potential impact on contamination. Thus, it was categorised as a high-risk behaviour. In contrast, parents sharing advice on social media was reported infrequently and appeared to have little potential for impacting behaviours. Thus, it was categorised as a low-risk behaviour.

We applied the contamination risk framework (Fig. 1) to develop strategies to both mitigate the risk of contamination in future trials through study design and develop strategies to monitor contamination during trial delivery. This will be applied to the future effectiveness trial and can also be applied to other similar complex intervention trials delivered in community based, public health settings (Table 2). For example, to mitigate the intervention group staff sharing information with the control group staff, the importance of minimising contamination should be explained during training to discourage sharing of intervention information. In terms of monitoring contamination, sharing of information between intervention arms by the staff could be recorded.

**Table 2:** Key findings, contamination risk and strategies for mitigating risk in future RCTs

Source of contamination	Implication	Strategies to mitigate contamination	Strategies to monitor contamination
**High risk (high chance, high impact)**			
Staff movement, as some staff worked across intervention and control children centres.	Some staff trained in intervention delivery shared HENRY messages at control sites through other programmes and when providing advice to parents.	• Ensure research setting is transparent about staff movement at the beginning of the study, so this can be considered during randomisation. • Ask staff not to share intervention content with control sites, and inform staff of the importance of reducing contamination so the importance of not sharing intervention content is understood • Deliver intervention outside of standard practice and as part of a research project. • Randomise at cluster level.	• Monitor staff movement at regular intervals
Staff had knowledge of the programme prior to the study, and all staff was briefed on the study including the HENRY programme. HENRY content was available to parents through children’s centre staff sharing advice and role modelling behaviours.	Some staff in control centres knew about the HENRY programme. Some staff made personal changes to be healthier, as a result of being involved in the study. This impacted on the information, advice and guidance given to parents, as this was based on personal experience.	• Provide training to centre staff on RCTs and the importance of minimising contamination. • Record any prior knowledge of intervention amongst staff. • keep control staff blinded as much as possible to intervention content • Randomise at cluster level.	
Staff meetings involved staff from control and intervention centres discussing best practice and programmes being run.	Some staff were aware that they should filter what was shared at these meetings, however found it challenging to do so. Some staff discussed sharing about intervention content to help staff at other centres. Staff routinely discussed programmes (including HENRY) at meetings.	• Encourage staff to not discuss the intervention at meetings, and to meet separately to discuss the programme with staff from the intervention arm only. • Inform staff of the importance of minimising contamination, so the importance of not sharing intervention content is understood.	• Report any sharing of information within meetings to research team.
**Medium- high risk (low chance/high impact)**			
Parents shared experiences of the HENRY programme with each other	The majority of parents did not attend/ have contact with parents from multiple centres and sharing was limited to parents who attend the same groups.	• Ask participants to not share intervention content and materials until after the study is completed.	• Asking parents to disclose what contact they have with other study centres or parents who attend other centres. • Add ‘Contamination questions’ to test parental understanding of programme content and thus, identify contamination.
Parents changed behaviour due to being recruited in study and aware of aim.	A few parents reported that they changed their behaviours once they had been recruited into the study as they knew that their weight was being monitored.	• Keep aim of study brief. • Promise intervention to control group once study is completed.	• Ask control participants if they have changed their behaviours due to being recruited into the study.
**Medium-low risk (high chance/low impact)**			
Parents shared existing content on social media.	Parents shared advice that other people/organisations had already posted on social media. Parents were unlikely to share about HENRY and usually shared articles or asked/ answered specific questions from other parents.	• Parents could be asked not to share about the intervention on social media for duration of study.	• Record information shared on social media.
**Low risk (low chance/low impact)**			
Some parents did not attend their local children’s centre and travelled further to attend one they preferred.	Parents did not have much contact with parents at other centres. If parents went to an intervention centre they may see HENRY displays/information.	• Do not encourage parents to attend other sites during study delivery.	• Monitor other centres that parents attend.
Children's centers sharing parenting advice on social media	Children’s centres used social media to promote activities as part of routine practice. No parents reported knowledge of HENRY via social media or had shared about it.	• Recommendation that only intervention centres post about programme if social media is used. • Recommend that if social media is to be used, social media posts promote the programme but do not share intervention content.	• Monitor if intervention facilitators share intervention information on social media.

## Discussion

Our nested qualitative research study within the HENRY feasibility study found that contamination was common and provided evidence that HENRY messages were shared between intervention and control centres. Sharing of public health messages is usually deemed as positive by intervention teams as a means to extend reach and potential impact; however, this sharing presents a challenge to researchers conducting trials within these settings as it is likely to reduce differences in outcome measures between the randomised groups. Previous work has statistically assessed information transfer to the control group or reviewed the strategies implemented in other RCTs to offer recommendations for researchers to reduce contamination [8–12]. In this study, we have qualitatively explored if and how contamination occurred, as well as the risk of the behaviour causing contamination to the control group. We have offered recommendations, which are supported by earlier work [10, 12].

We found that staff who had been trained to deliver HENRY reported working in control centres; many of whom acknowledged sharing HENRY advice to parents at control centres during the delivery of other programmes or if parents asked for advice. Allocating staff to work across multiple children’s centres or other community locations is a common used strategy to reduce costs and increase capacity [27]. It is recognised that this poses an increased risk of contamination [10, 11]. Where feasible, one solution would be to conduct cluster randomisation at a regional, rather than a children’s centre level. This would however result in larger cluster sizes, which could lead to underestimation of the intervention effectiveness if the number of clusters is not also increased [28]. If it is not feasible to cluster at a regional level, study designs could consider clustering centres that are geographically distinct to reduce the likelihood of messages being shared via staff or parents using more than one centre. Additionally, training of centre staff is recommended to enhance their understanding of the importance of restricting intervention messages to intervention centres only.

The results of our study demonstrated that the risk of contamination was relatively low amongst parents, who reported predominantly discussing information within children’s centres or within their immediate friend and family networks. However, investigating the extent and implications of contamination from a parental perspective was complex. Some participants found it challenging to recall specific examples of the source and impact of knowledge or advice they had received. Potentially, messages that had stemmed from HENRY may have been shared and not been acknowledged as a HENRY message. Future trials could prospectively monitor this by asking study participants within the control arm ‘blue dye’ questions during the trial. These ‘blue dye’ questions are able to test control group participants understanding of intervention content in order to identify if contamination may have occurred [10, 11].

Our findings were unable to quantify the extent to which contamination impacted behaviour, partly because of the inability of parents to recall specific details about the knowledge sharing. Thus, the presence of contamination may not necessarily always lead to behaviour change. This was largely influenced by the source of the information and frequency the information is received. Therefore, tracing of information crossover from control groups to intervention groups may not be sufficient, as conducted in earlier work [10, 12]. It is also important to identify if control parents changed their behaviours once they had received information.

In addition to challenges of recalling contamination, a further potential limitation was that staff may have withheld information about the extent to which they shared messages to the control arms. During recruitment to the feasibility study, centre managers received training which included the requirement to withhold discussion of HENRY outside of centres allocated to the HENRY trial arm. However, it is possible that this requirement was not shared amongst all centre staff, as many openly reported sharing knowledge. It is therefore recommended that future trials provide training regarding the trial protocol to all members of staff (i.e. not relying on information to filter down). Feedback obtained within the feasibility study process evaluation suggests that staff would welcome an online/electronic format for training if this is more feasible [Bryant et al., submitted at same time to Pilot and Feasibility Studies].

## Conclusions

Through an investigation of the sources and potential impact of contamination, this study has supported the design of a future effectiveness trial of HENRY, in addition to offering recommendations for future public health researchers to reduce the likelihood of contamination and monitor its presence during trial delivery (in order to mitigate or provide context to findings). Results indicate that centre staff is likely to pose the greatest risk of contamination within community based settings. Thus, clustering at a regional level may be appropriate in future research if feasible. Alternatively, strategies such as ensuring centre clusters are geographically distinct, and training all staff about the need to withhold knowledge from control centres is recommended. We recommend the implementation of our contamination risk framework and associated strategies to support the design of public health trials within community based settings. However, given the lack of evidence to quantify the risk of contamination influencing behaviour change, we also advocate research to explore this further. Importantly, the suggested strategies should not influence the ability of public health intervention teams to continue to share best practice.

## Data Availability

Original data is stored in a secure database within the University of Leeds. Scored and cleaned data, as well as output for analyses, are available upon request from the study PI, Maria Bryant

## Abbreviations

HENRY: Health Exercise and Nutrition for the Really Young
RCT: Randomised control trial
